# Probabilistic Clustering Using Multivariate Growth Mixture Model in Clinical Settings—A Scleroderma Example

**DOI:** 10.1002/sim.70450

**Published:** 2026-02-13

**Authors:** Ji Soo Kim, Yizhen Xu, Rachel S. Wallwork, Laura K. Hummers, Ami A. Shah, Scott L. Zeger

**Affiliations:** ^1^ Division of Rheumatology Johns Hopkins University School of Medicine Baltimore MD USA; ^2^ Division of Biostatistics, Department of Population Health Sciences University of Utah Salt Lake City UT USA; ^3^ Department of Biostatistics Johns Hopkins Bloomberg School of Public Health Baltimore MD USA

**Keywords:** Bayesian hierarchical models, multivariate growth mixture modeling, scleroderma, sequentially‐updating algorithm, trend‐based cluster membership

## Abstract

**Background:**

Scleroderma (systemic sclerosis; SSc) is a chronic autoimmune disease known for wide heterogeneity in patients' disease progression in multiple organ systems. Our goal is to guide clinical care by real‐time classification of patients into clinically interpretable subpopulations based on their baseline characteristics and the temporal patterns of their disease progression.

**Methods:**

A Bayesian multivariate growth mixture model was fit to identify subgroups of patients from the Johns Hopkins Scleroderma Center Research Registry who share similar lung function trajectories. We jointly modeled forced vital capacity (FVC) and diffusing capacity for carbon monoxide (DLCO) as pulmonary outcomes for 289 patients with SSc and anti‐topoisomerase 1 antibodies and developed a framework to sequentially update class membership probabilities for any given patient based on her accumulating data.

**Results:**

We identified a “stable” group of 150 patients for whom both biomarkers changed little from the date of disease onset over the next 10 years, and a “progressor” group of 139 patients that, on average, experienced a clinically significant decline in both measures starting soon after disease onset. For any given patient at any given time, our algorithm calculates the probability of belonging to the progressor group using both baseline characteristics and the patient's longitudinal FVC and DLCO observations.

**Conclusions:**

Our method calculates the probability of being a fast progressor at baseline when no FVC and DLCO are observed, then sequentially updates it as more information becomes available. This sequential integration of patient data and classification of her disease trajectory has the potential to improve clinical decisions and ultimately patient outcomes.

## 1 Introduction

Scleroderma (systemic sclerosis; SSc) is a multisystem autoimmune disease characterized by vasculopathy, dysregulation of the immune system, and exaggerated fibrosis [1]. It is well established that SSc patients demonstrate wide heterogeneity in clinical manifestations, speed and direction of disease progression, response to treatment, and survival [2, 3]. Severe organ complications, including interstitial lung disease (ILD), pulmonary arterial hypertension (PAH), cardiac involvement, and digital ulcers, occur in about 15% of patients [4]. Autoantibodies are known to associate with distinct phenotypes and risks of complications at a population level [5, 6], but significant variability in disease trajectory and the risk of major events is observed even within the same autoantibody strata at the patient level. By identifying unique temporal patterns in the progression of multiple biomarkers, we can gain a better understanding of the etiology of disease progression and make more informed treatment decisions.

Over the past decade, there has been a growing interest in developing methods to risk‐stratify patients into subgroups with similar progression patterns based on observed biomarkers, as these approaches may facilitate translational research studies focused on disease pathogenesis and inform targeted therapeutic approaches. Prior research on identifying clusters of SSc patients typically used a single outcome. When the outcome measure is continuous and roughly follows a Gaussian distribution, linear mixed effects models (LMM) [7] can flexibly describe patients' health trajectories over time. Latent class mixture models (LCMM), or growth mixture models (GMM) [8, 9], assume that the population comprises a small number of distinct classes across which the relationship of the outcome with fixed and random effects can vary. The index of the component indicates the membership of a patient in a specific cluster. LCMM assumes that there are a fixed number of latent clusters consisting of individuals with similar trajectories, where the unobserved individual‐level heterogeneity within each cluster is captured by cluster‐specific random effects. Group‐based trajectory modeling (GBTM) or Latent Class Growth Analysis (LCGA) [10] is a simplified model of LCMM that assumes all observations are independent of one another within a latent cluster and does not allow individual‐level random effects.

The LCGA model has gained popularity for identifying subgroups of biomarker trajectories in SSc patients due to its simplicity and low computational requirements. Using LCGA models, Barbacki et al. identified three trajectory groups using Scleroderma Clinical Trials Consortium Damage Index [11], a global irreversible damage score that combines multiple biomarkers, including respiratory measures. Man et al. [12] identified seven distinct trajectory patterns of percent predicted forced vital capacity (pFVC), and Kida et al. [13] identified five trajectory patterns of systolic pulmonary arterial pressure in a multicenter cohort study of SSc patients. After identifying the clusters, post‐hoc analyses were conducted to examine the relationship between patient characteristics and the odds of being in each trajectory cluster. The limitation of this strategy is that the cluster membership estimates can only be acquired for individuals in the dataset on which the model was trained. Additionally, the cluster discovery process does not account for known factors that influence the progression of trajectories. Schulam et al. [14] proposed hierarchical latent variable models to identify homogeneous SSc patient subgroups and separately modeled 4 continuous biomarkers (pFVC, total skin score, and percent predicted diffusing capacity of carbon monoxide (pDLCO), right ventricular systolic pressure). This approach enables flexible modeling of population, subgroup, and individual‐level longitudinal trajectories. However, when fitting the model separately for each measure, the number of subgroups identified across biomarkers varies. This variability makes it challenging to translate the results for clinical use, as patients' cluster memberships are then determined by combinations of cluster memberships specific to each measure.

Multivariate latent class mixture models (MLCMM) [15, 16] are better suited for identifying longitudinal clusters using multiple SSc biomarkers. Compared to LCMM, MLCMM enables the simultaneous modeling of multiple outcomes and addresses the between‐measure correlations using correlated random effects and random error terms. We introduce an MLCMM within a Bayesian framework. This method incorporates two key features: Firstly, it jointly analyzes the trajectories of pulmonary measures FVC and DLCO for subgroup discovery. Secondly, it allows for sequential updates of cluster membership estimates and trajectory predictions based on the dynamic accumulation of patient information. It is crucial to model both FVC and DLCO simultaneously since both biomarkers are strongly correlated. Failing to consider this association would lead to a decrease in the statistical efficiency of model estimation. Additionally, the dynamic forecast of patient condition using the MLCMM is crucial for the utility of such models in clinical settings.

A key premise of the analysis is that SSc patients can be separated into subgroups based upon both mechanistic and empirical evidence. We believe that disease progression can be predicted from known patient characteristics, such as autoantibody status, and from unobserved (latent) variables that are manifest in the patient's biomarker trajectories to date. If true, predictions for individuals can be improved by identifying subsets of people whose trajectories have similar temporal patterns not only in terms of baseline characteristics but also latent traits. The model we propose enables the efficient identification of underlying traits that simultaneously and flexibly characterize the temporal patterns of trajectory progression across multiple biomarker measures. The method not only enables the identification of patient subgroups based on their baseline status but also allows it to be updated with accumulating patient information.

The structure of this paper is as follows. We introduce the goal of the analysis and study cohort in Section 2.1 and define the multivariate pulmonary outcomes and their preprocessing in Section 2.2. The multivariate latent class mixture model framework and notations are in Section 2.3. Section 2.4 provides an application and details of the model specified to meet clinical aims using the Johns Hopkins Scleroderma Center Cohort data. The details of generating dynamic predictions utilizing the multivariate mixture model are in Section 2.5. We describe the data characteristics in Section 3.1, the selection process for determining the number of subgroups in Section 3.2, present the convergence diagnostics of model estimation in Section 3.3, and describe results of model fitting in Section 3.4. Section 4 includes a discussion on the advantages and potential clinical applications of the model, limitations of our method, and future research directions. In Section 5, we briefly highlight the clinical significance and summarize the main contributions of the approach.

## 2 Methods

### 2.1 Modeling Multivariate Longitudinal Outcome Measures

Our aim is to identify patient subgroups that represent distinct temporal patterns of pulmonary trajectories using Johns Hopkins Scleroderma Center Cohort patient data. For longitudinal outcome measures, we use pFVC and pDLCO among patients with systemic sclerosis. We jointly model the two measures using an MLCMM, where the correlation between them is addressed both on the patient level and in the stochastic errors. Our method not only enables the identification of patient groups based on their initial characteristics but also allows it to be updated with accumulating biomarker information. For this study, we limit our sample to 289 patients with anti‐topoisomerase 1 (topo) autoantibody, which is associated with a high risk of ILD. However, even within this subgroup, we observe heterogeneity in pulmonary function trajectory, which motivates our clinical aim.

### 2.2 Preprocessing Longitudinal Data

To ensure that the outcomes follow roughly Gaussian distributions prior to model fitting, we quantile‐normalized each measure. For each measure $Y_{k}$, where k=1,2 denotes pFVC and pDLCO, we obtain the quantile normalized values $\Phi^{-1}\circ {Ĝ}_{k}(Y_{k})$ where $\hat{G_{k}}$ is the estimated marginal distribution of $Y_{k}$ and $\Phi^{-1}$ is the inverse standard normal distribution. The quantile‐normalized values are used in model fitting, as described in the following sections.

### 2.3 Multivariate Latent Class Mixture Model

Assume the ith subject has $J_{i}$ measurement times and K outcomes $Y_{ij}=\{Y_{ij1},\ldots ,Y_{ijK}\}$ at each time $j\in \{1,\ldots ,J_{i}\}$. Let $U_{ijk}$ and $V_{ijk}$ be sets of covariates that are associated with disease progression; they may or may not be time‐varying. Specifically, we assume that $U_{ijk}$ affects population‐level trajectory and is shared across all latent subgroups, and $V_{ijk}$ is associated with systematic, unobserved heterogeneity in the latent disease progression. In other words, we hypothesize that the impact of $V_{ijk}$ on the overall progression of biomarkers in the population may differ systematically among unobserved subgroups. Without loss of generality, we assume no missingness in the data and that there are L mixture components. For subject i who belongs to the ℓth mixture component, we let $\mu_{ij}^{\left(\ell \right)}=(\mu_{ij1}^{\left(\ell \right)},\ldots ,\mu_{ijK}^{\left(\ell \right)})$ be the latent disease progression at time j. The MLCMM assumes that with the additional presence of measurement error $R^{\left(\ell \right)}$, there are K‐dimensional multivariate longitudinal outcomes that reflect the latent disease progression for each of the ℓth mixture component, i.e., $Y_{ij}^{\left(\ell \right)}=\{Y_{ij1}^{\left(\ell \right)},\ldots ,Y_{ijK}^{\left(\ell \right)}\}$. Note that the positive definite matrix $R^{\left(\ell \right)}\in {\mathbb{R}}^{K\times K}$ characterizes the correlation between measurement errors in $Y_{ij}^{\left(\ell \right)}$. For generality, we allow both the population average trends and correlation structures to differ between clusters. Define $C_{i}$ as the cluster membership of person i. In other words, if we know the true cluster membership $C_{i}$ (which cannot be directly observed), the model assumes that the observed outcome is

$$\begin{array}{l} \left(Y_{ij}\left|C_{i}=\ell ,Y_{ij}^{\left(1\right)},\ldots ,Y_{ij}^{\left(L\right)}\right.\right)=Y_{ij}^{\left(\ell \right)}. \end{array}$$

We assume that $\left\{\mu_{ijk}^{\left(\ell \right)};k=1,\ldots ,K\right\}$, the latent disease trajectory under $C_{i}=\ell$, follows the linear mixed effects model:

$$\begin{array}{l} \mu_{ijk}^{\left(\ell \right)}=U_{ijk}\beta_{Uk}+V_{ijk}\beta_{Vk}^{\left(\ell \right)}+Z_{ijk}b_{ik}^{\left(\ell \right)}, \end{array}$$

$$\begin{array}{l} \ell =1,\ldots ,L\;\text{and}\;j=1,\ldots ,J_{i}. \end{array}$$

For the kth biomarker, $\beta_{Vk}^{\left(\ell \right)}$ quantifies the effect of $V_{ijk}$ on the variation in the population‐level dynamic pattern of disease procedure, which is specific to the ℓth mixture component. In addition, $\beta_{Uk}$ represents the association between $U_{ijk}$ and the latent disease progression $\mu_{ijk}^{\left(\ell \right)}$, shared across all mixture components. Note that the distinct mixture components are largely characterized by systematically different patterns of the disease procedure in relation to $V_{ijk}$. The random effects $Z_{ijk}b_{ik}^{\left(\ell \right)}$ represent the individual‐level cluster‐specific deviation from the population average trajectories of the ℓth mixture components, $U_{ijk}\beta_{Uk}+V_{ijk}\beta_{Vk}^{\left(\ell \right)}$, where $Z_{ijk}\in {\mathbb{R}}^{p}$ are the subset of covariates that are associated with this heterogeneity. We assume that the random effect coefficients are distributed as $b_{i}^{\left(\ell \right)}=(b_{i1}^{\left(\ell \right)},\ldots ,b_{iK}^{\left(\ell \right)})∼MVN(\text{0},G^{\left(\ell \right)})$.

Denote $W_{i}$ as subject traits that provide information about the systematic unobserved heterogeneity characterized by cluster membership $C_{i}$. The cluster membership of person i, $C_{i}$, is modeled via logistic regression as follows, with cluster L as the reference level,

$$\begin{array}{ll} & \text{logit}[P(C_{i}=\ell |W_{i})]=\alpha_{0\ell }+W_{i}\alpha_{W\ell },\;\ell =1,\ldots ,L-1,\\ & P(C_{i}=L|W_{i})=1-\overset{L-1}{\underset{\ell =1}{\sum }}P(C_{i}=\ell |W_{i}), \end{array}$$

where $\alpha_{0\ell }$ be the intercept and $\alpha_{W\ell }$ is the coefficients for $W_{i}$. We define $\alpha_{0}=(\alpha_{01},\ldots ,\alpha_{0,L-1})$, $\alpha_{W}=(\alpha_{W1},\ldots ,\alpha_{W,L-1})$, and $\alpha =(\alpha_{0},\alpha_{W})$.

For the ℓth mixture component, $R^{\left(\ell \right)}$ and $G^{\left(\ell \right)}$ quantify the within and across‐biomarker covariance of the random errors and random effects, respectively. Conditional on $\left\{\mu_{ijk}^{\left(\ell \right)};k=1,\ldots ,K\right\}$, covariance matrices $R^{\left(\ell \right)}$ and $G^{\left(\ell \right)}$, and the cluster membership $C_{i}$, the observed outcomes $Y_{ij}$ are assumed to be independent across time and among patients. The distribution of biomarkers can then be written as

$$\begin{array}{lll} & \;f(Y_{i};\mu_{i},G,R)= & \overset{J_{i}}{\underset{j=1}{\prod }}\left[\overset{L}{\underset{\ell =1}{\sum }}P(C_{i}=\ell |W_{i})f\left(Y_{ij};\mu_{ij}^{\left(\ell \right)},G^{\left(\ell \right)},R^{\left(\ell \right)}\right)\right], \end{array}$$

where $Y_{i}=(Y_{i1},\ldots ,Y_{iJ_{i}})$, $\mu_{i}=\{\mu_{ij}^{\left(\ell \right)};j=1,\ldots ,J_{i},\ell =1,\ldots ,L\}$, $G=\{G^{\left(\ell \right)};\ell =1,\ldots ,L\}$, and $R=\{R^{\left(\ell \right)};\ell =1,\ldots ,L\}$.

### 2.4 Model Specification for the Johns Hopkins Scleroderma Center Cohort

The objective of developing our model using Johns Hopkins Scleroderma Center Cohort data is to meet the three clinical aims: (1) To identify distinct patterns of disease progression over time after adjusting for patients' baseline characteristics; (2) to generate an estimate of cluster membership for a new patient based on her baseline characteristics; and (3) to continuously update the estimated cluster membership and predict future biomarker trends using dynamically evolving and accumulating patient information. To efficiently address these three clinical aims, we jointly modeled the biomarkers to account for between‐measure correlations.

**FIGURE 1:**
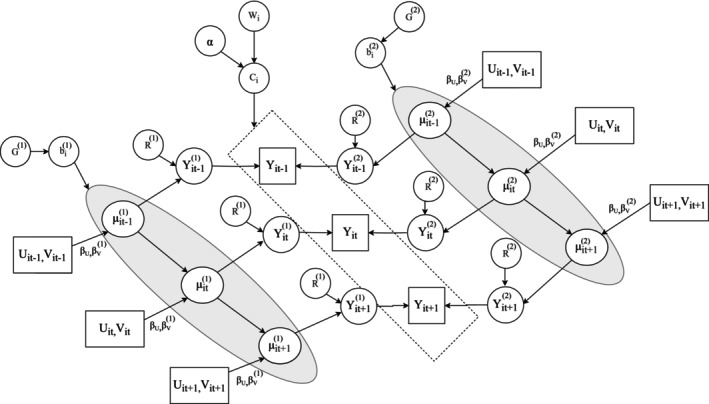
Directed acyclic graph under a simplified assumption of time‐invariant cluster membership. The arrows represent conditional dependencies. For example, the distribution of $Y_{ij}^{\left(\ell \right)}$ depends on $\mu_{ij}^{\left(\ell \right)}$ and $R^{\left(\ell \right)}$. Therefore, there are two arrows pointing from the latter variables to the former. Rectangles represent observed variables; ovals represent unobserved parameters or latent variables.

Figure 1 visualizes the dependency relationship among all the aforementioned parameters and variables. To simplify presentation, the figure assumes two mixture components and time‐invariant cluster membership. Furthermore, we define $V_{ijk}$ to be a smooth function of time such that the identified clusters will be characterized by unique longitudinal patterns of disease progression. The specification of $P(C_{i}|W_{i})$ in the MLCMM naturally serves as a baseline estimate of the cluster membership distribution, fulfilling the second clinical aim. Lastly, as illustrated in Section 2.5, the third clinical aim is addressed by using a Bayesian framework and employing posterior updating techniques to obtain real‐time calculations of cluster membership and cluster‐specific trajectory predictions.

After preprocessing pFVC and pDLCO, we model them jointly as a multivariate Gaussian distribution,

$$\begin{array}{l} \left(Y_{ij};\mu_{ij}^{\left(\ell \right)},G^{\left(\ell \right)},R^{\left(\ell \right)}\right)∼MVN\left(\mu_{ij}^{\left(\ell \right)},R\right), \end{array}$$

where we assumed the random errors in the observed outcomes have a shared covariance matrix R across different clusters. This assumption ensures that the clusters are defined by differences in the population‐level trajectory patterns and patient‐level deviations around them.

We specify the latent disease progression as follows:

$$\begin{array}{l} \mu_{ijk}^{\left(\ell \right)}=\beta_{0k}^{\left(\ell \right)}+b_{ik}^{\left(\ell \right)}+s_{ij}\beta_{1k}^{\left(\ell \right)}+X_{i}\beta_{2k}+s_{ij}X_{i}\beta_{3k}. \end{array}$$

Disease onset (t=0) is defined by the first non‐Raynaud's phenomenon (non‐RP) symptom. We denote $s_{ij}$ as the spline representation of the years since disease onset, $t_{ij}$, for the jth measurement of person i. To flexibly capture disease trajectories that vary over time, we used natural splines with 2 degrees of freedom with a knot at 5 years and boundary knots at 0 and 10 years (See Figure S1 in Supporting Information). These knot locations were chosen based on clinical knowledge of pulmonary disease progression in scleroderma. Variables that are known to influence lung trajectories from prior population studies are included as covariates ($X_{i}$) in the model. $X_{i}$ includes sex, self‐identified Black race, diffuse cutaneous subtype, and an indicator of later disease onset (≥50 years; median age of onset among 289 anti‐topo positive patients is 47). The same set of covariates is used for $W_{i}$, which explains subject cluster membership. We assume there is a shared trend across clusters that vary flexibly by $X_{i}$, defined by $\beta_{2k}$ and $\beta_{3k}$. Cluster‐specific deviation from this shared mean trend is characterized by $\beta_{0k}^{\left(\ell \right)}$, $\beta_{1k}^{\left(\ell \right)}$, and individual‐level deviation from the cluster‐specific trajectory is captured by a random intercept $b_{ik}^{\left(\ell \right)}$. Details of prior specifications are in Appendix A. For model estimation, we use 2000 burn‐in samples before $N_{post}=3000$ posterior draws and run the estimating procedure using the package *rstan* [17] in the R software. We determined the number of latent subgroups by using Bayesian information criterion (BIC) [18] and checked model convergence by using the potential scale reduction factor (PSRF) [19].

### 2.5 Dynamic Predictions

To address the last clinical aim, we developed algorithms to (1) continuously update the probability of belonging to the fast progressor group ($p_{it}$) for patient i and at time t given her past measures of both pFVC and pDLCO; and (2) generate a dynamic projection of an individual's future trajectories based on their history. In our model, cluster membership $C_{i}$ is conceptualized as a time‐invariant, unobserved latent trait specific to each individual. While $C_{i}$ itself does not vary over time, our Bayesian framework enables dynamic updating of its posterior distribution as more longitudinal data become available. Specifically, we compute the posterior probability of cluster membership at time t using the observed data up to that point via:

$$p_{it}=P(C_{i}=\ell |Y_{i,1:t};\beta ,\alpha ,G,R),$$

which provides a time‐varying estimate of the fixed latent cluster assignment. This reflects an evolving belief about $C_{i}$, rather than a change in the cluster membership itself. We propose a computational scheme for the dynamic calculation of $p_{it}$ in Appendix B. To separately project the person's disease progression in both the fast progressor and stable groups, we sample from the posterior predictive distribution of $Y_{i,t+1}^{\left(1\right)}$ for the fast progressor group and $Y_{i,t+1}^{\left(2\right)}$ for the stable group, following the computational scheme described in Appendix C. For example, the posterior predictive distribution of $Y_{i,t+1}^{\left(\ell \right)}$ would be represented by $N_{post}$ posterior samples $\left\{Y_{i,t+1}^{\left[1](\ell \right)},\ldots ,Y_{i,t+1}^{\left[N_{post}](\ell \right)}\right\}$, with ℓ being 1 for the fast progressor group and 2 for the stable group.

## 3 Results

### 3.1 Data Characteristics

A total of 289 anti‐topo positive SSc patients were selected for this analysis by the following inclusion criteria: pFVC and pDLCO values collected within 10 years since the onset of the first non‐RP symptom, patients with at least 3 pFVC and pDLCO values in this 10‐year window, and patients whose first pFVC or pDLCO is observed within the first 5 years since non‐RP onset. We use complete data with no missingness in both the covariates and longitudinal outcomes. The dataset consists of 2329 observations, and the number of observed outcomes per patient ranges from 3 to 28, with a median of 7 observations. The upper part of Table 1 displays a summary of the variables.

**TABLE 1:** Data summary stratified by the identified trajectory clusters: The fast progressor and stable groups.

	All (N = 289)	Fast progressor (*N* = 139)	Stable (*N* = 150)
	Mean (SD) or # (%)	Mean (SD) or # (%)	Mean (SD) or # (%)
Male	47 (16.3)	19 (13.7)	28 (18.7)
Self‐identified Black race	65 (22.5)	46 (33.1)	19 (12.7)
Diffuse	165 (57.1)	98 (70.5)	67 (44.7)
Age of onset (non‐RP)	46.3 (13.6)	51.1 (13.1)	41.8 (12.5)
Baseline pFVC	80.8 (18.4)	79 (17.5)	82.6 (19)
Baseline pDLCO	67.1 (22.6)	63.6 (22.2)	70.4 (22.5)

	Mean (SD) or # (%)	Mean (SD) or # (%)	Mean (SD) or # (%)
Disease duration (non‐RP)	1.5 (1.2)	1.4 (1.1)	1.6 (1.3)
Disease duration	3.9 (6)	3.7 (6.6)	4.1 (5.5)
Age at baseline	47.8 (13.6)	52.6 (13.2)	43.4 (12.4)
Anti‐Centromere	3 (1)	3 (2.2)	0 (0)
Anti Fibrillarin	8 (3)	2 (1.6)	6 (4.3)
Anti Ku	15 (5.7)	9 (7.3)	6 (4.3)
Anti NOR90	7 (2.7)	5 (4.1)	2 (1.4)
Anti PMScl	0 (0)	0 (0)	0 (0)
Anti RNAPol	5 (1.9)	2 (1.5)	3 (2.1)
Anti Ro52	34 (12.9)	18 (14.6)	16 (11.4)
Anti Th/To	19 (7.2)	8 (6.5)	11 (7.9)
Anti U1RNP	40 (15.6)	23 (19.2)	17 (12.5)
Baseline ILD	28 (30.4)	17 (35.4)	11 (25)
Baseline pFVC < 70	80 (27.7)	42 (30.2)	38 (25.3)
ILD (Ever)	233 (81.8)	120 (87.0)	113 (76.9)
ILD at 1 year from baseline	92 (41.1)	52 (46.8)	40 (35.4)
Baseline PH	8 (6.5)	7 (11.1)	1 (1.6)
PH (Ever)	93 (33.2)	61 (44.5)	32 (22.4)

*Note:* The variables involved in model estimation are summarized in the upper table. For continuous variables, we provide the mean (standard deviation), while for binary variables, we provide the count (percent positive). The lower table examines important clinical variables and their relationship with the identified trajectory clusters using the same summary scheme. The autoantibodies represent autoantibody overlap in patients who are anti‐topo positive. Disease duration is calculated by taking the difference between disease onset (the earlier of Raynaud's and non‐RP symptoms) and baseline. ILD is ascertained by HRCT or X‐ray, and baseline ILD has a missing rate of 68.2%. Pulmonary hypertension (PH) is defined as RVSP ≥45 mmHg.

### 3.2 Estimation of Number of Trajectory Subgroups

**FIGURE 2:**
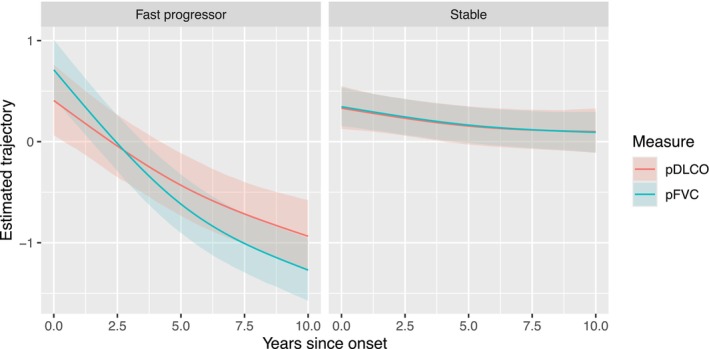
Estimated average trajectories of pFVC and pDLCO in a standardized scale for each identified cluster among anti‐topo positive patients in the reference subgroup: Non‐diffuse, non‐Black, female patients with early onset. The values are quantile‐normalized so that observations within each measure follow the standard normal distribution. The solid line represents the posterior mean, while the confidence band represents the 95% credible interval of the population‐level temporal patterns. The fast progressor group (left) consists of patients with faster progressing trajectories of pFVC and pDLCO, and the stable group (right) consists of patients with stable trajectories for both measures. The coefficient estimates used to generate these trajectories are shown in Figure 3.

The model with two trajectory subgroups had the lowest BIC of 4124.8, compared to other models with one and three clusters for which BICs were 4478.4 and 4136.6, respectively. Therefore, we consider the two‐subgroup model as the optimal choice under the assumed framework. We identified a “stable” group of 150 patients for whom both biomarkers changed little from the date of disease onset over the next 10 years (Figure 2 right panel), and a “progressor” group of 139 patients, which on average experienced a steep decline in both measures starting soon after disease onset (Figure 2 left panel). Our method allows us to visualize the expected subgroup‐specific lung trajectories for any combination of the baseline factors under consideration.

### 3.3 Convergence Diagnostics

We use the PSRF to demonstrate that the posterior estimation of model parameters under our assumed two‐subgroup model achieved sufficient Markov chain Monte Carlo (MCMC) convergence. The PSRF is a consistent estimator of Monte Carlo variance and is a Gelman–Rubin convergence diagnostic for MCMC. The estimate of the multivariate PSRF is 1.00016 for cluster‐specific and shared fixed effects coefficients $\left(\beta_{U},\beta_{V}^{\left(1\right)},\beta_{V}^{\left(2\right)}\right)$, 1.00113 for the cluster‐specific covariance matrices of random effects $\left(G^{\left(1\right)},G^{\left(2\right)}\right)$, 0.99995 for the shared covariance matrix of the random errors, R, and 1.00148 for the coefficients in the baseline probability of fast progressors $\left(\alpha_{0},\alpha_{W}\right)$. Given that the multivariate PSRF is close to 1 when the samples have converged to the target distribution, we conclude that the posterior samples from the estimated model demonstrate sufficient convergence.

### 3.4 Model Estimation Results

In the upper part of Table 1, we summarized the variables used in the model stratified by the two identified groups. For example, 47 patients in the dataset are male, constituting 16.3% of the data population, and the estimation procedure classified 19 of them into the fast progressor group and the remaining 28 males into the stable group. The age of disease onset defined by the first non‐RP symptom has an overall mean of 46.3, while the mean age of onset is 51.1 and 41.8 in the fast progressor and stable groups, respectively. In the lower part of Table 1, we further studied the relationship between the identified subgroups and other variables that may have scientific relevance but were not included in the model. We observe that within the topo autoantibody subset, most autoantibodies do not demonstrate a large discrepancy between the identified clusters, except for anti‐U1RNP, where there are 23 and 17 patients in the fast progressor and stable groups, respectively.

**FIGURE 3:**
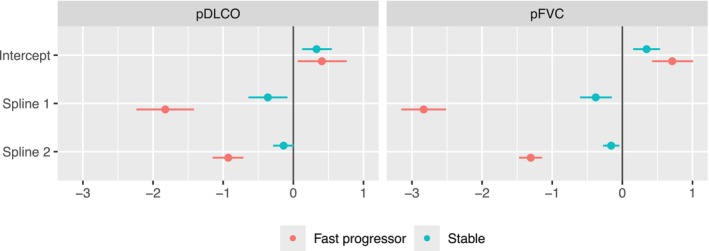
Posterior mean and 95% credible interval of the estimated coefficients of the cluster‐specific time trends that describe the progression of pFVC and pDLCO trajectories. Spline 1 and Spline 2 are obtained from natural cubic splines, with a knot at 5 years and bounded at 0 and 10 years.

**FIGURE 4:**
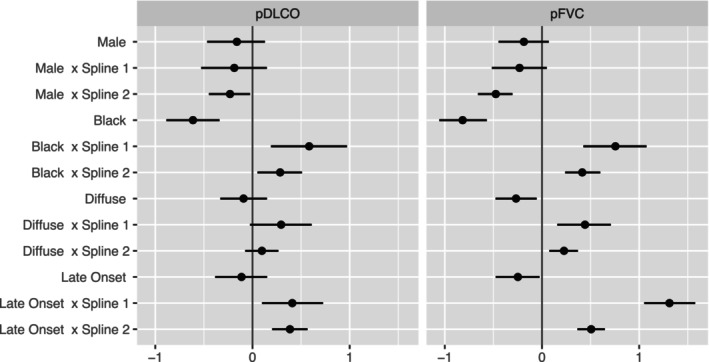
Posterior mean and 95% credible interval of the estimated coefficients of the shared factors and their interactions with time trends (Spline 1 and Spline 2, obtained from natural cubic splines, with a knot at 5 years and bounded at 0 and 10 years) that describe the progression of pFVC and pDLCO trajectories.

Figure 3 summarizes $\left(\beta_{0k}^{\left(1\right)},\beta_{1k}^{\left(1\right)}\right)$ and $\left(\beta_{0k}^{\left(2\right)},\beta_{1k}^{\left(2\right)}\right)$, and Figure 4 summarizes $\left(\beta_{2k},\beta_{3k}\right)$, where k=1 and 2 corresponds to pFVC and pDLCO, respectively. The estimated values and the corresponding 95% credible intervals are presented in Table S1 of the Supporting Information. Specifically, Figure 2 visualizes the population‐level temporal trends in the two identified subgroups, which is equivalent to setting $\left(\beta_{2k},\beta_{3k}\right)$ to zero. This describes the estimated trends for female, non‐Black, and non‐diffuse patients with disease onset before they are 50 years old. The fast progressor group has a rapid deterioration of pulmonary measures compared to the stable group, which has a significantly slower progression. We present the estimated posterior predictive distribution for the cluster‐specific random‐effects covariances $\left(G^{\left(1\right)},G^{\left(2\right)}\right)$, the shared covariance matrix of the random errors, R, and the coefficients for the variables in the baseline probability of being in the fast progressor group, $\left(\alpha_{0},\alpha_{W}\right)$ in Table S2 of Supporting Information. On average, patients whose baseline characteristics are self‐identified Black race, diffuse cutaneous subtype, and later disease onset tend to have a significantly higher chance of being fast progressors, i.e., a faster deterioration in pulmonary measures.

**FIGURE 5:**
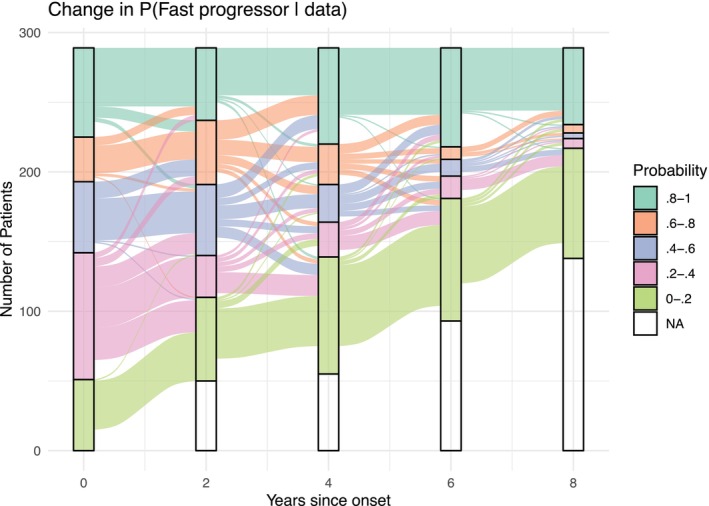
Flowchart demonstrating changes in the probability of being in the fast progressor cluster of 289 anti‐topo positive patients. NA (white space) indicates no probability estimated due to pFVC or pDLCO not observed. The estimated probabilities at baseline (0 years since first visit) are more evenly distributed across the 5 probability groups, indicating there is low certainty of group membership for most patients. As more pFVC and pDLCO data are observed, estimated probabilities are closer to 0 or 1 for most patients, demonstrating an increase in certainty.

At time 0, when no pFVC and pDLCO values are observed, the probability of being in the fast progressor group, $p_{i0}$, is calculated based on only baseline clinical and demographic characteristics. We obtain more precise estimations of individualized health trajectories and $p_{it}$ as more longitudinal observations become available. Figure 5 displays the dynamic change in the posterior probability of belonging to the fast progressor group over the years since disease onset across all patients in the observed dataset. The diagram presents the posterior probability in 5 intervals, from between 0 and 0.2 to between 0.8 and 1, representing an increasing likelihood of rapid pulmonary progression. As the follow‐up time increases in the flowchart, the proportion of individuals with a risk between 0.2 and 0.8 decreases. This is because our confidence in distinguishing between progressors and stable patients increases as we accumulate more observations per patient. By the eighth year, the algorithm had reached a high level of certainty regarding the classification of the patients.

**FIGURE 6:**
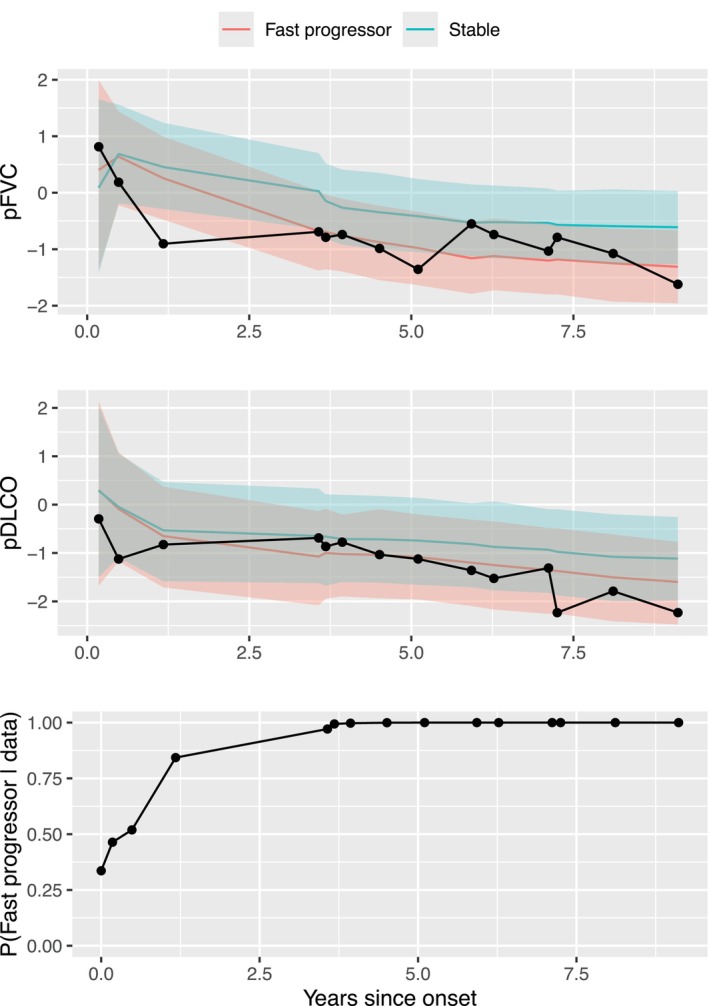
Observed and estimated pFVC and pDLCO trajectories in a standardized scale by group membership and estimated probabilities of belonging to the fast progressor group. A randomly selected patient's normalized observed pFVC (top) and pDLCO (middle) are shown in black, and her sequentially updated estimated trends (solid lines) and their 95% credible intervals (shades) are shown in blue and pink, given that the person is in the fast progressor or stable group, respectively. The estimated probabilities of belonging to the fast progressor group given available observed data are also sequentially updated after each visit (bottom). The first point shown in the bottom plot (at 0 year since onset) is the probability of being a fast progressor given only baseline information (without any pFVC or pDLCO measurement), that is $\exp(\alpha_{01}+W_{i}\alpha_{W1})/\{\sum_{\ell =1}^{2}\alpha_{0\ell }+W_{i}\alpha_{W\ell }\}$ where ℓ=1 corresponds to the fast progressor group. The estimated probability of being in the fast progressor group is close to 1 after observing pFVC and pDLCO around 1 year since onset.

In Figure 6, we present a real‐time projection of pFVC and pDLCO trajectories and their 95% credible intervals for a randomly selected individual. This figure is an illustration of our proposed dynamic prediction schemes described in Section 2.5. The selected patient is female, non‐Black, has diffuse cutaneous disease, and was 42 years old when she developed SSc. At baseline (t=0), when no observations of pFVC or pDLCO were available, the estimated probability of belonging to the fast progressor group is 0.34, an estimate entirely based on her baseline covariates. After the first three observations of pFVC and pDLCO, the probability increases to 0.84 and stays close to 1 for the rest of the visits. We also see that her projected pFVC and pDLCO trajectories as a fast progressor represent the pattern in her observed data better than those of the stable group.

## 4 Discussion

There is substantial clinically relevant variability in disease progression among SSc patients as manifested in both biomarkers and risk of major clinical events. Autoantibodies serve as useful clinical tools for risk stratification, yet even within an autoantibody subgroup, significant heterogeneity remains. There is still a huge unmet need to discover homogeneous subgroups of patients that share similar disease features, underlying mechanisms, complication risks, and therapeutic responses. In our work, we developed a modeling framework to identify latent subgroup(s) of patients who share similar progression patterns of multiple biomarkers.

Our model is designed to quantify risk factors of progression in pulmonary measures and to provide real‐time predictions for individual patients of cluster membership and future trajectories at the point of care. The model assumes that baseline characteristics, including age of onset, sex, race, and cutaneous subtype, affect both the cluster membership and the disease trajectories. Beyond these known factors, the model also assumes that there are unmeasured factors that make some patients stable and others progress more rapidly. The goal of the model is to identify subgroup(s) that correspond to different temporal patterns in the biomarkers and to use the data observed for a given patient for classifying her into the more likely progression pattern.

Among anti‐topo patients, we used a multivariate latent class mixture model to segregate progressors from those who are likely to remain stable. The subgroups are identified by the joint trajectories of the two measures, pFVC and pDLCO, while allowing for cluster‐specific correlation structures between them. This is crucial in our setting, as the two biomarkers are known to be biologically correlated, and accounting for their joint evolution while allowing the correlation structure to vary across latent subgroups enables more accurate characterization of unmeasured patient heterogeneity. To our knowledge, fitting models with this level of flexibility is not feasible in existing likelihood‐based implementations of multivariate growth mixture models. Rather than providing an estimated cluster membership for each measure per patient, we assume that there is one underlying individual‐specific latent class reflected jointly by multiple biomarker trajectories. This assumption leads to more clinically useful and relevant results, particularly because pFVC and pDLCO have a strong correlation. Additionally, employing such a joint modeling strategy results in more statistically efficient inferences. Reference Kim et al. [20] quantified the inefficiency of modeling biomarkers separately as compared to jointly modeling them in a combined model. In the motivating clinical case study of scleroderma trajectories with five biomarkers, including pFVC and pDLCO, they demonstrated that greater efficiency gains in estimating random effects are expected in the case of high across‐measure correlation.

Further, an important feature of our approach is the sequential updating of class membership probabilities and individual‐level predictions as new data accumulate over time. This dynamic, personalized updating is essential for precision medicine applications, where treatment or monitoring decisions depend not only on a patient's baseline characteristics, but also on evolving risk or trajectory profiles. Implementing this coherently within a Bayesian framework allows us to incorporate uncertainty and borrow strength across patients and time points. We observed that the certainty of belonging to the fast progressor or stable group increases as the observations of pFVC and pDLCO accumulate over time. This approach enables us to utilize past information to enhance prediction beyond the estimated model parameters, even for patients whose cluster membership was largely uncertain at baseline. This type of updating is not readily achievable under standard likelihood‐based estimation approaches, which typically assume a fixed dataset and do not naturally support real‐time re‐estimation without retraining the full model.

The future direction of this research is to discover novel biomarkers that can effectively distinguish individuals with rapid disease progression from those with slow or moderate progression. Biomarkers that can differentiate these patient subgroups will provide valuable insight into the etiology of the disease and serve as useful tools for risk assessment. Identifying these biomarkers can yield crucial information that helps medical decision‐making, including determining which patients may potentially receive greater benefits from intensive monitoring and therapeutic strategies, such as combined immunosuppressive treatments. For future research, we plan to investigate the association of rapid progression in pulmonary function with molecular and immunological biomarkers for SSc patients in the early stages of the disease.

Our analysis has several limitations. Because we were interested in studying heterogeneity within a high‐risk subgroup, we focused the study population on individuals who tested positive for anti‐topo antibody. The analysis only included observations of pFVC and pDLCO when both biomarkers were collected simultaneously. Our future work involves expanding the analysis to include all patients in the Johns Hopkins Scleroderma Center Research Registry. Additionally, we will refine the model likelihood to handle missing data more efficiently. Further research can be done to broaden the model's scope by integrating additional types of biomarkers, such as genetic, molecular, and immunological biomarkers, and by developing appropriate variable selection methodology. Lastly, our model does not account for the influence of treatment on the progression of biomarkers over time, which could potentially affect the identification of subgroups. Our model assumes that cluster membership remains constant over time, representing latent patient traits. The probability of a person belonging to each cluster is updated depending on observed longitudinal measurements. In future work, we will address this limitation by defining time‐invariant cluster membership as a function of immunosuppressive medications to account for the distinct temporal patterns in how patients respond to treatment. Additionally, we can continue to adopt the subgroup definition in this proposal and incorporate the treatment effect by assuming that cluster membership is time‐varying.

## 5 Conclusion

We developed a practical tool that satisfies the clinical need of identifying latent subgroups of SSc patients defined based on the temporal patterns of their pulmonary measures. We proposed a Bayesian framework utilizing MLCMM that can provide a probabilistic estimate of the initial cluster membership for new patients. Our method also allows time‐evolving predictions of future biomarker trajectories and cluster membership based on patients' accumulating longitudinal observations. We implemented our method on data from anti‐topo positive patients in the Johns Hopkins Scleroderma Center Cohort and discovered two subgroups that correspond to the commonly observed patient types known as “fast progressors” and “stable” as referred to by clinicians. Our tool can be flexibly adapted to find distinct progression patterns based on other clinically important longitudinal biomarkers, from which we can discover novel biomarkers that associate with the identified disease progression patterns.

## Funding

This work was supported in part by the NIH/NIAMS under Grant Nos. P30AR070254, R01AR073208, and K24AR080217, and in part by the Jerome L. Greene Foundation, Rheumatology Research Foundation, Johns Hopkins inHealth initiative, the Scleroderma Research Foundation, the Nancy and Joachim Bechtle Precision Medicine Fund for Scleroderma, the Manugian Family Scholar, the Donald B. and Dorothy L. Stabler Foundation, the Chresanthe Staurulakis Memorial Fund, and the Sara and Alex Othon Research Fund.

## Ethics Statement

The authors have nothing to report.

## Conflicts of Interest

The authors declare no conflicts of interest.

## Supporting information




**Data S1**: sim70450‐sup‐0001‐Supinfo.pdf.

## Data Availability

Data are available upon reasonable request. The data are from the Johns Hopkins Scleroderma Center Research Registry that spans several decades, with different consent forms used over time. The data cannot be made publicly available since public data sharing was not covered under the original consent forms. Deidentified participant data are available upon reasonable request through the Registry PI (Ami Shah, email Ami.Shah@jhmi.edu, ORCID: 0000‐0002‐1139‐2009) with appropriate IRB approval and inter‐institutional data sharing agreements between the requester's institution and Johns Hopkins University. All code used to implement the proposed methodology and to perform the analyses is provided as Supporting Information and is also available at https://github.com/yizhenxu/Clustering‐SSc‐Trajectories.

## Appendix A Prior Specifications

We use non‐informative priors for model parameters:

$$\begin{array}{ll} & \beta_{Uk},\beta_{Vk}^{\left(\ell \right)},\alpha_{0\ell },\alpha_{W\ell }∼N(0,100),\\ & G^{\left(\ell \right)}∼\text{inverse‐Wishart}(\nu_{G},S_{G}),\\ & R^{\left(\ell \right)}∼\text{inverse‐Wishart}(\nu_{R},S_{R}), \end{array}$$

The hyperparameters $\left(\nu_{G},S_{G}\right)$ and $\left(\nu_{R},S_{R}\right)$ are the degrees of freedom and scale matrix for the priors of G and R, respectively, where $S_{G},S_{R}\in {\mathbb{R}}^{K\times K}$. By the properties of inverse‐Wishart, the prior mean and mode of G are $\frac{S_{G}}{\nu_{G}-K-1}$ and $\frac{S_{G}}{\nu_{G}+K+1}$, respectively. To have proper prior, $\nu_{G}$ must satisfy $\nu_{G}>K+1$. For the application, the degree of freedom is chosen as the smallest allowable integer value such that the inverse‐Wishart prior distributions are the most diffuse while guaranteeing that G and R have proper priors. We set $\nu_{G}=K+2$ such that the prior mean of G is $S_{G}$. The same consideration applies to R. Thus, $\nu_{G}=\nu_{R}=4$ and $S_{G}=S_{R}={ℐ}_{2}$. The resulting prior mean is ${ℐ}_{2}$ and the prior mode is ${ℐ}_{2}\times 1/7$ for both G and R.

## Appendix B Sequential Update of Cluster Probability

For an ith person, assume there are K biomarkers, and without loss of generality, assume there is no missingness. Write observed outcomes for this ith person as

$$\begin{array}{ll} & \;Y_{i,1:t}=(Y_{i1,1:K}^{T},Y_{i2,1:K}^{T},\ldots ,Y_{it,1:K}^{T})\\ = & {\left(Y_{i11},\ldots ,Y_{i1K},Y_{i21},\ldots ,Y_{i2K},\ldots ,Y_{it1},\ldots ,Y_{itK}\right)}^{T}. \end{array}$$

The target quantity is the time‐varying estimate of cluster membership,

$$\begin{array}{ll} & \;P(\text{Cluster}\;\ell |Y_{i,1:t};\beta ,\alpha ,G,R)\\ = & \frac{P(C_{i}=\ell |\alpha_{\ell })P(Y_{i,1:t}|\text{Cluster}\;\ell ;\beta ,\alpha ,G,R)}{\overset{L}{\underset{c=1}{\sum }}P(C_{i}=c|\alpha_{c})P(Y_{i,1:t}|\text{Cluster}\;c;\beta ,\alpha ,G,R)}, \end{array}$$

ℓ=1,2, where

$$\begin{array}{ll} & \;P(Y_{i,1:t}|\text{Cluster}\;c;\beta ,\alpha ,G,R)\\ = & \int P(Y_{i,1:t}|\text{Cluster}\;c,b_{i}^{\left(c\right)};\beta ,\alpha ,G,R)P(b_{i}^{\left(c\right)};G)db_{i}^{\left(c\right)}. \end{array}$$

Define vector of random effects $b_{i}^{\left(c\right)}=(b_{i1}^{\left(c\right)},\ldots ,b_{iK}^{\left(c\right)})$ and vector of parameters in the membership model for the ℓth cluster, $\alpha_{\ell }=(\alpha_{0\ell },\alpha_{W\ell })$. In detail, each term can be further expressed as

$$\begin{array}{ll} & \;P(Y_{i,1:t}|\text{Cluster}\;c,b_{i}^{\left(c\right)};\beta ,\alpha ,G,R)\\ = & \overset{t}{\underset{j=1}{\prod }}\frac{1}{{\left(2\pi \right)}^{K/2}|R{|}^{1/2}}\times \\ & \exp\left\{-\frac{1}{2}{\left(Y_{ij,1:K}-\mu_{ij,1:K}^{\left(c\right)}\right)}^{T}R^{-1}(Y_{ij,1:K}-\mu_{ij,1:K}^{\left(c\right)})\right\} \end{array}$$

and

$$\begin{array}{ll} & \;P(b_{i}^{\left(c\right)};G^{\left(c\right)})\\ & =\overset{K}{\underset{k=1}{\prod }}\frac{1}{{\left(2\pi \right)}^{K/2}|G^{\left(c\right)}{|}^{1/2}}\exp\left\{-\frac{1}{2}b_{i}^{(c)T}{\left(G^{\left(c\right)}\right)}^{-1}b_{i}^{\left(c\right)}\right\}. \end{array}$$

By the Gaussian distribution assumptions and the independence between ϵ and $\mu_{ijk}^{\left(c\right)}$'s,

$$\begin{array}{ll} & \;P\left(Y_{i,1:t}\left|\text{Cluster}\;c;\beta ,\alpha ,G^{\left(c\right)},R\right.\right)\\ = & \overset{t}{\underset{j=1}{\prod }}\frac{1}{{\left(2\pi \right)}^{K/2}|G^{\left(c\right)}+R{|}^{1/2}}\times \exp\left\{-\frac{1}{2}\times \right.\\ & \left.{\left(Y_{ij,1:K}-g_{ij,1:K}^{\left(c\right)}\right)}^{T}{\left(G^{\left(c\right)}+R\right)}^{-1}\left(Y_{ij,1:K}-g_{ij,1:K}^{\left(c\right)}\right)\right\} \end{array}$$

where $g_{ijk}^{\left(c\right)}=\beta_{0k}^{\left(c\right)}+s_{ij}\beta_{1k}^{\left(c\right)}+X_{i}\beta_{2k}+s_{ij}X_{i}\beta_{3k}$.

## Appendix C Sequential Update of Future Trajectories

The goal is to sample $b_{i}^{\left(c\right)}$ conditional on observed outcomes $Y_{i,1:t}$ for the prediction of $Y_{i,t+1}$. Define $1_{t}={\left(1,\ldots ,1\right)}^{T}\in {\mathbb{R}}^{t}$ as the length‐t vector of ones, and let $I_{t}$ and $I_{K}$ denote the identity matrices of dimensions t×t and K×K, respectively. Define $Z_{itK}=1_{t}⊗I_{K}\in {\mathbb{R}}^{tK\times K}$. Note that the Kronecker product $I_{t}⊗R\in {\mathbb{R}}^{tK\times tK}$. We start with the joint distribution of $b_{i}^{\left(c\right)}$ and $Y_{i,1:t}$,

$$\begin{array}{ll} & P(Y_{i,1:t},b_{i}^{\left(c\right)}|\text{Cluster}\;c,b_{i}^{\left(c\right)};\beta ,\alpha ,G,R)∼\\ & MVN\left(\left(\begin{array}{c} g_{i,1:t}^{\left(c\right)}\\ 0 \end{array}\right),\left(\begin{array}{cc} Z_{itK}G^{\left(c\right)}Z_{itK}^{T}+I_{t}⊗R & Z_{itK}G^{\left(c\right)}\\ G^{\left(c\right)}Z_{itK}^{T} & G^{\left(c\right)}, \end{array}\right)\right) \end{array}$$

where $g_{i,1:t}^{\left(c\right)}={\left(g_{i11}^{\left(c\right)},\ldots ,g_{i1K}^{\left(c\right)},\ldots ,g_{it1}^{\left(c\right)},\ldots ,g_{itK}^{\left(c\right)}\right)}^{T}$. As a result, we can obtain the conditional distribution of the random effects,

$$\begin{array}{ll} & P(b_{i}^{\left(c\right)}|Y_{i,1:t};\beta ,\alpha ,G,R)∼\\ & MVN\left(G^{\left(c\right)}Z_{itK}^{T}{\left[Z_{itK}G^{\left(c\right)}Z_{itK}^{T}+I_{t}⊗R\right]}^{-1}\left(Y_{i,1:t}-g_{i,1:t}^{\left(c\right)}\right),\right.\\ & \left.G^{\left(c\right)}-G^{\left(c\right)}Z_{itK}^{T}{\left[Z_{itK}G^{\left(c\right)}Z_{itK}^{T}+I_{t}⊗R\right]}^{-1}Z_{itK}G^{\left(c\right)}\right) \end{array}$$

Given $\beta^{\left[s\right]},\alpha^{\left[s\right]},G^{\left[s\right]},R^{\left[s\right]}$ from the sth posterior draw of model estimation, we get the corresponding posterior prediction of the outcome $Y_{i,t+1}=(Y_{i,t+1,1},\ldots ,Y_{i,t+1,K})$ conditional on $g_{i,t+1,k}^{\left[s](\ell \right)}=\beta_{0k}^{\left[s](\ell \right)}+s_{i,t+1}\beta_{1k}^{\left[s](\ell \right)}+X_{i}\beta_{2k}^{\left[s\right]}+s_{i,t+1}X_{i}\beta_{3k}^{\left[s\right]}$, ℓ=1,2, by the following steps:

1. sample $b_{i}^{\left[s](\ell \right)}∼P(b_{i}^{\left(\ell \right)}|Y_{i,1:t};\beta^{\left[s\right]},\alpha^{\left[s\right]},G^{\left[s\right]},R^{\left[s\right]}).$
2. calculate cluster‐specific predictions
  $$\begin{array}{l} Y_{i,t+1}^{\left[s](\ell \right)}∼MVN\left(b_{i}^{\left[s](\ell \right)}+g_{i,t+1}^{\left[s](\ell \right)},R^{\left[s\right]}\right), \end{array}$$
  where $g_{i,t+1}^{\left[s](\ell \right)}=(g_{i,t+1,1}^{\left[s](\ell \right)},\ldots ,g_{i,t+1,K}^{\left[s](\ell \right)})$.
3. obtain $\lambda^{\left[s](\ell \right)}=P(C_{i}=\ell |Y_{i,1:t};\beta^{\left[s\right]},\alpha^{\left[s\right]},G^{\left[s\right]},R^{\left[s\right]})$ via the procedure in the previous section.
4. predict $Y_{i,t+1}^{\left[s\right]}$ by sampling from $\left(Y_{i,t+1}^{\left[s](1\right)},\ldots ,Y_{i,t+1}^{\left[s](L\right)}\right)$ based on multinomial distribution with probability $\left(\lambda^{\left[s](1\right)},\ldots ,\lambda^{\left[s](L\right)}\right)$.

As a result, we obtain samples $\left\{Y_{i,t+1}^{\left[s\right]}|s=1,\ldots ,N_{post}\right\}$ as representing the posterior predictive predictions of $Y_{i,t+1}$.
