# Supplementary material for: Probabilistic Clustering Using Multivariate Growth Mixture Model in Clinical Settings—A Scleroderma Example
Source: Stat Med. 2026 Feb 13;45(3-5):e70450. doi: 10.1002/sim.70450 (PMC12904757; doi:10.1002/sim.70450)
Supplement: Supplementary file 1 — Data S1: sim70450‐sup‐0001‐Supinfo.pdf. [file SIM-45-0-s001.pdf]

Supplementary Materials for "Probabilistic Clustering using Multivariate Growth Mixture Model in Clinical Settings - A Scleroderma Example"

August 11, 2025

1 Spline Visualization

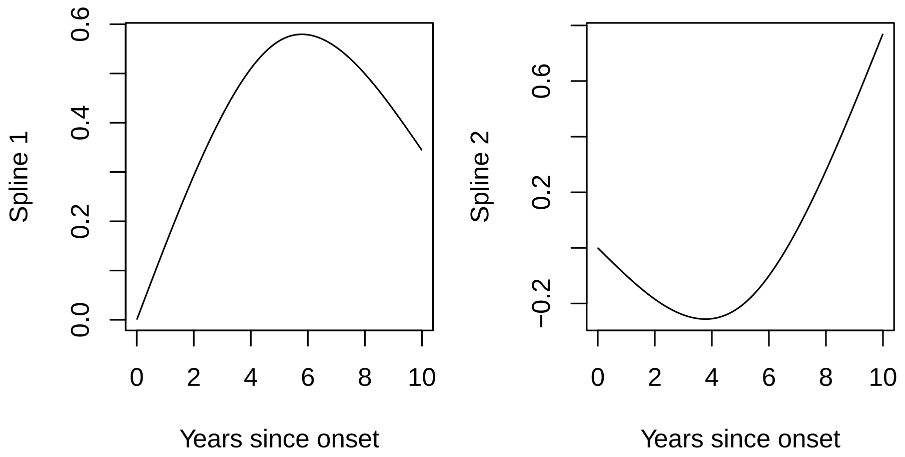

Figure 1: Visualization of Spline 1 and Spline 2 obtained under natural spline transformation of years since onset.

2 Estimation of Coefficients

Cluster	Variable	pFVC	pDLCO
Fast progressor	Intercept	0.71 (0.42, 1.01)	0.41 (0.06, 0.76)
	Spline 1	-2.83 (-3.15, -2.51)	-1.83 (-2.24, -1.42)
	Spline 2	-1.31 (-1.47, -1.15)	-0.93 (-1.15, -0.71)
Stable	Intercept	0.35 (0.15, 0.54)	0.33 (0.13, 0.55)
	Spline 1	-0.38 (-0.6, -0.15)	-0.36 (-0.64, -0.08)
	Spline 2	-0.16 (-0.27, -0.04)	-0.14 (-0.29, 0.01)
Shared	Male	-0.19 (-0.45, 0.07)	-0.16 (-0.47, 0.13)
	Male x Spline 1	-0.23 (-0.52, 0.05)	-0.19 (-0.53, 0.15)
	Male x Spline 2	-0.48 (-0.66, -0.3)	-0.23 (-0.45, -0.02)
	Self-identified Black race	-0.82 (-1.06, -0.57)	-0.61 (-0.89, -0.34)
	Self-identified Black race x Spline 1	0.76 (0.43, 1.08)	0.58 (0.19, 0.97)
	Self-identified Black race x Spline 2	0.41 (0.24, 0.6)	0.28 (0.05, 0.51)
	Diffuse	-0.27 (-0.48, -0.05)	-0.09 (-0.33, 0.15)
	Diffuse x Spline 1	0.44 (0.16, 0.71)	0.29 (-0.03, 0.61)
	Diffuse x Spline 2	0.23 (0.08, 0.37)	0.1 (-0.07, 0.27)
	Late onset	-0.25 (-0.48, -0.03)	-0.11 (-0.38, 0.15)
	Late onset x Spline 1	1.31 (1.05, 1.58)	0.41 (0.1, 0.73)
	Late Onset x Spline 2	0.51 (0.37, 0.65)	0.39 (0.2, 0.57)

Table 1: Posterior mean and 95% credible interval of the estimated coefficients for both shared factors and cluster-specific time trends that describe the progression of pFVC and pDLCO trajectories. Years since onset is coded as natural cubic splines, with a knot at 5 and bounded at 0 and 10 years.

Cluster	Variable	Mean (95% CI)
Fast progressor	$G_{11}^{(1)}$	0.49 (0.38, 0.62)
	$G_{12}^{(1)}$	0.34 (0.24, 0.47)
	$G_{22}^{(1)}$	0.6 (0.46, 0.77)
Stable	$G_{11}^{(2)}$	0.59 (0.45, 0.75)
	$G_{12}^{(2)}$	0.38 (0.26, 0.53)
	$G_{22}^{(2)}$	0.62 (0.47, 0.81)
Shared R	R_{11}	0.1 (0.09, 0.1)
	R_{12}	0.05 (0.04, 0.05)
	R_{22}	0.19 (0.17, 0.2)
Shared Latent Coefficients	Intercept	-2.23 (-3.12, -1.39)
(α_0, α_W)	Male	-0.76 (-1.85, 0.26)
	Black	1.61 (0.75, 2.55)
	Diffuse	1.54 (0.73, 2.38)
	Late Onset	2.41 (1.68, 3.24)

Table 2: Posterior mean and 95% credible interval of the coefficients in the baseline probability of being in the fast progressor group, (α_0, α_W), and the estimated covariance matrices for the cluster-specific random effects ($G^{(1)}$ for fast progressor group and $G^{(2)}$ for stable group) and the random errors that is shared between the two clusters (R). The ith row and jth column entry is denoted by R_{ij} for R, $G_{ij}^{(\ell)}$ for $G^{(1)}$ and $G^{(2)}$.

3 Variables of Scientific Relevance

	Mean (SD) or #(%), Missing(%)		
Age of onset (rp)	43.8 (14.1), 2.77	48.5 (13.7), 4.32	39.5 (13), 1.33
Age of onset	43.5 (14), 2.77	48.3 (13.7), 4.32	39.2 (12.9), 1.33
Disease duration (nonrp)	1.5 (1.2), 0	1.4 (1.1), 0	1.6 (1.3), 0
Disease duration (rp)	3.7 (6.2), 2.77	3.4 (6.7), 4.32	3.9 (5.6), 1.33
Disease duration	3.9 (6), 2.77	3.7 (6.6), 4.32	4.1 (5.5), 1.33
Age at baseline	47.8 (13.6), 0	52.6 (13.2), 0	43.4 (12.4), 0
Anti Centromere	3 (1), 0.35	3 (2.2), 0	0 (0), 0.67
Anti Fibrillarin	8 (3), 9	2 (1.6), 11.51	6 (4.3), 6.67
Anti Ku	15 (5.7), 9	9 (7.3), 11.51	6 (4.3), 6.67
Anti NOR90	7 (2.7), 9	5 (4.1), 11.51	2 (1.4), 6.67
Anti PMScl	0 (0), 9	0 (0), 11.51	0 (0), 6.67
Anti RNAPol	5 (1.9), 6.57	2 (1.5), 6.47	3 (2.1), 6.67
Anti Ro52	34 (12.9), 9	18 (14.6), 11.51	16 (11.4), 6.67
Anti Th/To	19 (7.2), 9	8 (6.5), 11.51	11 (7.9), 6.67
Anti U1RNP	40 (15.6), 11.42	23 (19.2), 13.67	17 (12.5), 9.33

Table 3: This table examines important clinical variables and their relationship with the identified trajectory clusters. For continuous variables, we provide the mean (standard deviation), while for binary variables, we provide the count (percent positive among non-missing entries), with an additional summary of the percentage of missing entries. The autoantibodies represent autoantibody overlap in patients who are anti-topo positive. Disease duration is calculated by taking the difference between disease onset (the earlier of Raynaud's and non-RP symptom) and baseline.

4 Code for Model Estimation

```
setwd("S:/TrajectoryAnalysis/STAN") # set working directory

source("dataprocessing3_0-10_scl70.R") # load data

fn <- "mod.stan" # stan file name to save as
fitname <- "mod2fit_sharedBeta_230813_scl70_functionalTheta.RData" # fitted model saved into this

scode = "
data {
  int<lower=0> Nsubs; //number of subjects

  int<lower=0> NpredsX; // ncol of design matrix X

  int<lower=0> NpredsS; // ncol of design matrix S

  int<lower=0> Nobs; //number of non-NA observations
  int<lower=1, upper=Nsubs> subject[Nobs]; // created fake ID 1,..., Nsubs

  matrix[Nobs,2] Y; //  outcomes matrix
  matrix[Nobs,NpredsX] X; // design matrix X cluster-specific
  matrix[Nobs,NpredsS] S; // design matrix S shared by clusters

  int<lower=0> L; // number of mixture components, 2
  int<lower=0> nb; //number of random effects, 2

  real Gnu; // hyperparameters for sampling G
  matrix[2,2] GS;

  real Rnu; // hyperparameters for sampling R
  matrix[2,2] RS;

  int lsub[Nsubs]; //element i being total number of time points for person i
  int endidx[Nsubs]; // cumulative_sum(lsub);

  matrix[Nsubs, 5] Z;
}
parameters {
  cov_matrix[2] R; // variance of error term epsilon

  matrix[nb, Nsubs] z_1;
  matrix[2, NpredsX] beta_1;
  cov_matrix[nb] G_1; // covariance of b's

```

```
  matrix[nb, Nsubs] z_2;
  matrix[2, NpredsX] beta_2;
  cov_matrix[nb] G_2;

  vector[NpredsS] betaS[2]; //beta for S

  vector[5] alpha;
}
transformed parameters {

  simplex[2] theta[Nsubs];

  matrix[Nsubs, nb] b_1; // b0(Y1), b1(Y1), b0(Y2), b1(Y2)
  matrix[nb,nb] sqrG_1;
  matrix[Nobs,2] mu_1; // X * beta + Z * b

  matrix[Nsubs, nb] b_2;
  matrix[nb,nb] sqrG_2;
  matrix[Nobs,2] mu_2;

  sqrG_1 = cholesky_decompose(G_1);
  b_1 = (sqrG_1 * z_1)'; // b[n] ~ N(0, L*L') = N(0, G)
  for(n in 1:Nobs){
    mu_1[n,1] = S[n] * betaS[1] + X[n] * beta_1[1]' + b_1[subject[n],1];
    mu_1[n,2] = S[n] * betaS[2] + X[n] * beta_1[2]' + b_1[subject[n],2];
  }

  sqrG_2 = cholesky_decompose(G_2);
  b_2 = (sqrG_2 * z_2)';
  for(n in 1:Nobs){
    mu_2[n,1] =  S[n] * betaS[1] + X[n] * beta_2[1]' + b_2[subject[n],1];
    mu_2[n,2] =  S[n] * betaS[2] + X[n] * beta_2[2]' + b_2[subject[n],2];
  }

  for(i in 1:Nsubs){
    theta[i,1] = inv_logit(Z[i] * alpha);
    theta[i,2] = 1 - theta[i,1];
  }

}
model {

  to_vector(z_1) ~ normal(0,1);
```

```
  to_vector(z_2) ~ normal(0,1);

  betaS[1] ~ normal(0,10);
  betaS[2] ~ normal(0,10);
  alpha ~ normal(0,10);

  to_vector(beta_1) ~ normal(0,10); // Gaussian(0, var = 100)
  G_1 ~ inv_wishart(Gnu, GS);

  to_vector(beta_2) ~ normal(0,10);
  G_2 ~ inv_wishart(Gnu, GS);

  R ~ inv_wishart(Rnu, RS);


  for(i in 1:Nsubs){
    vector[L] lps = log(theta[i]);
      for(n in (endidx[i]-lsub[i]+1):endidx[i] ){
        lps[1] += multi_normal_lpdf(Y[n] | mu_1[n], R);
        lps[2] += multi_normal_lpdf(Y[n] | mu_2[n], R);
      }//Ni
      target += log_sum_exp(lps);
  }//i

}
generated quantities {
  matrix[Nsubs, L] logpyz;
  real log_lik = 0;

  for(i in 1:Nsubs){
    vector[L] lps = log(theta[i]);
      for(n in (endidx[i]-lsub[i]+1):endidx[i] ){
        lps[1] += multi_normal_lpdf(Y[n] | mu_1[n], R);
        lps[2] += multi_normal_lpdf(Y[n] | mu_2[n], R);
      }//Ni
      for(l in 1:L){
        logpyz[i,l] = lps[l];
      }//L
    log_lik += log_sum_exp(lps);
  }//i


}
"

datalist = list(Nsubs = max(refdat$ID),
```

```
                 Nobs = nrow(xY), NpredsX = ncol(xY), NpredsS = ncol(xS),
                 subject = refdat$ID, Y = Yvec,
                 X = xY, S = xS,
                 nb = 2, L = 2, Gnu = 4, Rnu = 4, GS = diag(2), RS = diag(2),
                 lsub = lsub, endidx = cumsum(lsub),
                 Z = Z)


parvec = c("betaS","beta_1","beta_2","G_1","G_2","b_1","b_2","R", "alpha", "log_lik", "logpyz")

# Delete existing mod.stan
if (file.exists(fn))  file.remove(fn)

# Write Stan file
writeLines(scode, fn)
rstan:::rstudio_stanc(fn)

# Run Stan model
nit = 5000
nwup = 2000
seednum = 1
nc = 10 # number of chains

options(mc.cores = nc) #parallel::detectCores()
fit = stan(file = fn, data = datalist,
           pars = parvec,
           include = T, chains = nc, iter = nit, warmup = nwup, thin = 1, seed = seednum)

save(fit, file  = fitname)

# traceplot - log posterior
traceplot(fit, pars = "lp__", inc_warmup = F)
```
